# Health Insurance Coverage and Impact: A Survey in Three Cities in China

**DOI:** 10.1371/journal.pone.0039157

**Published:** 2012-06-18

**Authors:** Kuangnan Fang, BenChang Shia, Shuangge Ma

**Affiliations:** 1 Department of Statistics, School of Economics, Xiamen University, Xiamen, China; 2 Department of Statistics and Information Science, FuJen Catholic University, New Taipei City, Republic of China; 3 School of Public Health, Yale University, New Haven, Connecticut, United States of America; Tehran University of Medical Sciences, Iran (Islamic Republic of)

## Abstract

**Background:**

China has one of the world's largest health insurance systems, composed of government-run basic health insurance and commercial health insurance. The basic health insurance has undergone system-wide reform in recent years. Meanwhile, there is also significant development in the commercial health insurance sector. A phone call survey was conducted in three major cities in China in July and August, 2011. The goal was to provide an updated description of the effect of health insurance on the population covered. Of special interest were insurance coverage, gross and out-of-pocket medical cost and coping strategies.

**Results:**

Records on 5,097 households were collected. Analysis showed that smaller households, higher income, lower expense, presence of at least one inpatient treatment and living in rural areas were significantly associated with a lower overall coverage rate. In the separate analysis of basic and commercial health insurance, similar factors were found to have significant associations. Higher income, presence of chronic disease, presence of inpatient treatment, higher coverage rates and living in urban areas were significantly associated with higher gross medical cost. A similar set of factors were significantly associated with higher out-of-pocket cost. Households with lower income, inpatient treatment, higher commercial insurance coverage, and living in rural areas were significantly more likely to pursue coping strategies other than salary.

**Conclusions:**

The surveyed cities and surrounding rural areas had socioeconomic status far above China's average. However, there was still a need to further improve coverage. Even for households with coverage, there was considerable out-of-pocket medical cost, particularly for households with inpatient treatments and/or chronic diseases. A small percentage of households were unable to self-finance out-of-pocket medical cost. Such observations suggest possible targets for further improving the health insurance system.

## Introduction

In 2007, the WHO ranked China's health system as 144^th^ in terms of quality and access out of 190 countries, far below poorer countries like Haiti [1]. The discrepancy between economic advancement and health system development had motivated the Chinese government to undertake a system-wide reform of its health sector [2]. According to the Chinese Healthcare Reform Plan, an important component of the health sector reform is the reform of health insurance, with the main goal to make it more accessible and more affordable [3].

The health insurance system in China is composed of basic health insurance and commercial health insurance. The basic health insurance is run by the central and local governments. It consists of three schemes for different groups of people and takes different forms in rural and urban China. In rural China, the new rural cooperative medical care system (NCMS) was designed as a mutual help and risk-pooling health protection system [4], [5], [6]. It is government-led and running on a voluntary basis. Premium contributions are subsidized by the central and local governments, while contributions from the insured are relatively limited. Patients can get reimbursed for about 75% of the medical expenses between the deductible and the ceiling. As reported by the central government, by 2010, the number of participants of NCMS had reached 835 million, accounting for 96.3% of the total rural population [7]. In urban China, there are two primary programs, namely the urban employee basic medical insurance for urban employed (UEBMI) and urban resident basic medical insurance for urban residents (URBMI). UEBMI requires the enrollment of all urban employees. Employers and employees share the responsibility of paying premium contributions. URBMI mainly covers urban residents who are not officially employed as well as children. The pooling fund is mainly used for hospitalization fees and medical expenses incurred in outpatient clinics for critical diseases, however, doesn't cover usual outpatient care [8]. Combined together, China's basic medical insurance is providing coverage for more than 1.25 billion people [7]. At present, the Ministry of Health is responsible for NCMS. Basic health insurance in the urban regions is under the umbrella of the Ministry of Human Resources and Social Security.

Commercial health insurance is supplementary to basic health insurance, targeting mainly the upper class [8]. Chinese commercial insurers are supervised by the China Insurance Regulatory Commission (CIRC). According to CIRC, commercial health insurance accounted for about 6.03% of all medical insurance premiums and 5.72% of all insurance reimbursements in 2008 [8].

In the literature, one of the most relevant studies is [2], which provided a comprehensive review of the evolvement of the health care system. The policy aspect of reforming China's urban health insurance was discussed in [3]. An empirical study of coverage and assessment of the reform was conducted using survey data in [9]. Recently, more and more attention has been paid to the health insurance system in rural regions. A review of NCMS was provided in [6]. Lei and Lin [10] studied the service and health outcome aspects of NCMS. Qiu and others [11] studied the rural to urban migration and its impact on NCMS. Wang and others [12] discussed the adverse selection problem in NCMS. Published studies may have the following limitations. First, although they provided valuable insights at the time of publication, they can be outdated at present. The health insurance system in China has been undergoing a system-wide reform with fast progress [6], [7], [8]. Second, most published studies focused on the basic health insurance. A considerable percentage of Chinese population are now covered by both basic and commercial health insurance [13]. From the perspectives of coverage, impact of illness conditions and coping strategies, it is not sensible to separate the two insurance systems and focus only on the basic insurance. Third, most studies had been conducted in rural areas. China is undergoing fast urbanization. It was estimated that by the end of 2010, the mainland of China had a total urban population of 665.57 million, 49.68% of the total population [14]. Thus, urban areas deserve equal attention as rural areas.

The study design strategy is as follows. To provide an up-to-date description, a survey was conducted. To complement existing studies, the survey was focused on three major cities and surrounding areas. To provide a more comprehensive description, both basic and commercial insurances were studied. Our analysis strategy is as follows. As a broad coverage is needed for health insurance to be effective, our first set of analysis focused on coverage. A main objective of health insurance was to reduce cost for the insured. Thus, in the second set of analysis, we studied the cost, both gross and out-of-pocket, as they have different implications. As considerable cost might still exist after insurance reimbursement, the third set of analysis was on how households dealt with such cost. The three sets of analyses are closely linked.

## Methods

### Data Collection

The survey was conducted by staff at the Data Mining Center, Xiamen University. A Computer-Assisted Telephone Survey System (CATSS) was used. The collection of phone numbers for survey was obtained from China Telecom Corp. Ltd. and China Unicom Corp. Ltd. The following method was used for RDD (random digit dialing) selection of samples. We draw Mitofsky-Waksberg [15] type samples of active blocks of 100 consecutive phone numbers from all possible such blocks within each city. The probability of a block's initial selection was proportional to the block's 100 numbers that served residences. The study database was updated constantly to ensure that no household was sampled twice. As “cell phone only” households remained low and it was difficult to associate a cell phone number with a physical location, we focused on landline only.

At the beginning of each phone call, the survey staff would gather basic information. A household would be excluded if (1) the interviewee refused to participate, or (2) it was not in the three surveyed cities (defined by “Hukou”), or (3) the interviewee was less than eighteen years old, or (4) the interviewee could not provide reliable information on the household (self-evaluation). Verbal consent was obtained from each interviewee and recorded using a voice recording software package. A total of fifteen questions were asked. Some questions, for example household size and insurance coverage, were “snapshots” at the time of survey. Other questions, for example household income, expense, illness conditions and coping strategies, reflected the accumulation over twelve months. On average, one interview lasted five minutes.

### Statistical Analysis

Data was de-identified prior to analysis. We examined data and found no obviously unreasonable measurements. In this study, we were interested in three aspects of health insurance. The first was coverage. Here we analyzed overall coverage as well as basic and commercial insurance coverage separately. For each household, we dichotomized coverage rates at 50% and created dummy variables. The second aspect was medical cost, including gross medical cost (before insurance reimbursement) and net out-of-pocket medical cost (after insurance reimbursement). In the survey, medical cost was classified into five categories (<1K, 1K≤ <3K, 3K≤ <5K, 5K≤ <10K, 10K≤, all in Chinese Yuan). The third aspect was coping. Strategies for dealing with high and extremely high cost were analyzed separately. We examined the differences between data collected in difference cities using ANOVA and Chi-squared tests and determined that it was appropriate to combine data. As the response variables of interest were categorical, logistic regression was the main analysis tool. Model diagnostics was conducted, and no serious deviation from the model assumption was detected. All analysis was conducted using S-Plus Version 8.2 (TIBCO Software Inc).

## Results and Discussion

### Survey

A survey was conducted by staff at the Data Mining Center, Xiamen University, China, in July and August, 2011. The study was approved by a research ethics review committee at Xiamen University. Three major cities, Beijing, Shanghai and Xiamen, and their surrounding rural areas were included in the survey (Table 1). Beijing and Shanghai were chosen as they are the two most important cities in China. Xiamen was chosen as a representative city in the southeastern area. The three cities represent major cities with a relatively higher socioeconomic status (in 2010, the per capita GDP for the whole China was $4,382). The study collected data on 5,097 households (Table 2). The survey response rate was about 48%. In Asian tradition, household had been the functional unit for income and expense [16]. As an important goal of this study was to investigate the financial impact of health insurance, data was collected and analyzed at the household level.

**Table 1:** Description of the three surveyed cities.

	City		
	Beijing	Shanghai	Xiamen-Zhangzhou
Location	Northern, Coastline	Middle, Coastline	Southeast, Coastline
Population*	19.6 million	23 million	5 million
GDP, per capita* +	$10,672	$11,134	$9,438
Municipality	Yes	Yes	No

* As of 2010.

+ At the time of survey, $1 USD = 6.37 Yuan.

**Table 2:** Basic characteristics of all subjects and stratified by insurance status.

Variable	Total	Overall coverage		Basic insurance coverage		Commercial insurance coverage	
		>50%	≤50%	>50%	≤50%	>50%	≤50%
Sample Beijing Shanghai Xiamen	5097 1578 1530 1989	4437 1380 1342 1715	660 198 188 274	4154 1294 1258 1602	943 284 272 387	977 319 308 350	4120 1259 1222 1639
P value		(0.363)		(0.368)		(0.074)	
Household size Mean (sd)	3.706 (1.520)	3.666 (1.559)	3.976 (1.196)	3.633 (1.576)	4.029 (1.192)	2.896 (1.599)	3.898 (1.435)
P value		(<0.001)		(<0.001)		(<0.001)	
**Household income (Percentage)**							
Less than 30K	23.76	23.28	26.97	23.42	25.24	29.17	22.48
30K–50K	23.29	21.57	34.85	20.73	34.57	17.09	24.76
50K–100K	25.23	26.14	19.09	26.38	20.15	20.06	26.46
100K–150K	15.81	16.50	11.21	17.02	10.50	15.46	15.90
More than 150K	11.91	12.51	7.88	12.45	9.54	18.22	10.41
P value		(<0.001)		(<0.001)		(<0.001)	
**Household expense (Percentage)**							
Less than 10K	12.11	11.52	16.06	10.74	18.13	15.05	11.41
10K−30K	30.23	28.92	39.09	28.60	37.43	22.93	31.97
30K–50K	31.53	32.43	25.45	33.25	23.97	25.08	33.06
50K–100K	16.77	17.35	12.88	17.53	13.47	18.63	16.33
More than 100K	9.36	9.78	6.52	9.89	7.00	18.32	7.23
P value		(<0.001)		(<0.001)		(<0.001)	
**Number of inpatient treatment (Percentage)**							
None	73.02	74.74	61.52	75.49	62.14	77.69	71.92
One	17.99	16.11	30.61	15.67	28.21	16.07	18.45
Two	6.34	6.56	4.85	6.07	7.53	4.09	6.87
Three	1.24	1.42	0	1.52	0	0	1.53
Four	0.16	0.18	0	0.19	0	0.82	0
Five or more	1.26	0.99	3.03	1.06	2.12	1.33	1.24
P value		(<0.001)		(<0.001)		(<0.001)	
**Presence of chronic diseases (Percentage)**							
Yes	25.11	25.4	23.18	24.84	26.3	23.44	25.51
No	74.89	74.6	76.82	75.16	73.7	76.56	74.49
P value		(0.240)		(0.374)		(0.193)	
**Hukou (Percentage)**							
Urban	71.3	73	59.85	73.38	62.14	76.25	70.12
Rural	28.7	27	40.15	26.62	37.86	23.75	29.88
P value		(<0.001)		(<0.001)		(<0.001)	

* Values in “()” are p-values of Chi-squared or Fisher's exact test.

### Sample characteristics

Summary statistics on coverage were first computed. The 5,097 households contained 18,889 members, among which 15,555 (82.35%) and 6,568 (34.77%) were covered by basic and commercial insurance, respectively. 1,147 households had full, 27 had zero, and the rest had partial coverage. With a high overall coverage rate, it was interesting to notice that a very small number of households had no coverage at all. This small group deserves special attention in future studies. In the literature, there were many studies on analyzing percentage data. However, our literature review suggested that none was able to properly accommodate data with “two non-ignorable corners” (nonzero percentages of zeros and ones). We dichotomized the coverage ratio at 50%, and studied the contrast of “majority” versus “minority with coverage”. Out of the 5,097 households, 4,437 (87.05%) had more than 50% of the household members covered. 4,154 (81.50%) and 977 (19.17%) households had more than 50% of the members covered by basic and commercial health insurance, respectively. The calculated basic insurance coverage rate was lower than that provided by the central government but higher than that reported in [17].

Household summary statistics were computed for the whole cohort and subgroups generated based on insurance coverage rates (Table 2). We conducted between-group comparisons using t-test, Chi-squared test or Fisher's exact test, depending on data characteristics. Smaller households tended to have higher coverage rates. The average household sizes were 3.666 and 3.976 for the high and low coverage groups (p-value for difference <0.001), respectively. There was a significant difference in income between groups with different coverage rates (p-value from Chi-squared test <0.001). Particularly, households with higher income tended to have more coverage. For example, in the income <30 K group ($1 USD = 6.37 Yuan), 85.30% households had coverage rate over 50%; As a comparison, in the income >150 K group, 91.43% had coverage rate over 50%. As expense is tightly connected to income, we observed a similar association between coverage rates and expense. In the survey, there were two measures of household health conditions. The first was the number of hospitalized inpatient treatments, which was a surrogate for high-cost, low-frequency health shocks. The second measure was the presence of members with chronic diseases, which was a measure of low-cost, high-frequency health shocks. There was a significant association between coverage and presence of inpatient treatment. 38.48% households in the low coverage group had at least one inpatient treatments. As a comparison, 25.26% households in the high coverage group had at least one inpatient treatments (p-value from Chi-squared test <0.001). However, no significant association was observed between the presence of chronic diseases and coverage rate. We surveyed both urban and surrounding rural areas. The rural versus urban status was defined based on “Hukou”, which was a central government-issued ID card for the whole household. 71.3% of the total households were in the urban areas. We observed a significant association between coverage rates and Hukou. Particularly, urban residents had relatively higher coverage rates.

Summary statistics suggested that household size, income, expense, health conditions and location of household were potentially associated with coverage and financial impact of health insurance and warranted further analysis. This set of variables was comparable to those in published studies [16], [18], [19].

### Analysis of coverage

In the coverage analysis (Table 3), results from univariate and multivariate analysis were mostly consistent. Multivariate analysis suggested that bigger households had significantly lower overall coverage (odds ratio 0.893), higher basic insurance coverage (odds ratio 4.500) and lower commercial insurance coverage (odds ratio 0.558). For overall and basic insurance coverage, the “between 30 K and 50 K” income group had significantly lower coverage compared with the reference group (<30 K). For commercial insurance, three higher income groups had significantly lower coverage (odds ratios 0.634, 0.628 and 0.693, respectively, all p-values<0.001). Compared with the reference group (<10 K), two higher expense groups (between 30 K and 50 K, and between 50 K and 100 K) had significantly higher overall coverage. For basic insurance, all expense groups differed significantly from the reference group. However, there was no linear relationship between expense and coverage (test for linearity p-value<0.001). For commercial insurance, the “between 10 K and 30 K” and “over 100 K” groups were significantly different from the reference group. Presence of chronic disease was positively associated with a higher basic insurance coverage rate, suggesting possible selection effects, as chronic diseases are long lasting, allowing households with presence of chronic diseases to pursue basic insurance to cope with future medical expense. Households with inpatient treatments had lower overall and basic insurance coverage (odds ratios 0.541 and 0.533, respectively, p-values<0.001). No significant difference between cities was observed.

**Table 3:** Coverage rate (>50%): univariate and multivariate logistic regressions.

	Overall		Basic	Commercial		
	Univariate	Multivariate	Univariate	Multivariate	Univariate	Multivariate
Household size	0.880 (<0.001)	0.893 (<0.001)	0.849 (<0.001)	4.500 (<0.001)	0.571 (<0.001)	0.558 (<0.001)
Income (baseline: <30K) B: between 30K and 50K C: between 50K and 100K D: between 100K and150K E: over 150K	0.717 (0.002) 1.586 (<0.001) 1.704 (<0.001) 1.839 (<0.001)	0.599 (<0.001) 1.207 (0.209) 1.275 (0.155) 1.246 (0.289)	0.646 (<0.001) 1.411(0.001) 1.747 (<0.001) 1.405 (0.010)	0.856 (<0.001) 0.449 (0.210) 0.847 (0.758) 1.048 (0.097)	0.532 (<0.001) 0.584 (<0.001) 0.749 (0.010) 1.349 (0.008)	0.634 (<0.001) 0.628 (0.001) 0.693 (0.013) 0.899 (0.510)
Expense (baseline: <10K) B: between 10K and 30K C: between 30K and 50K D: between 50K and 100K E: over 100K	1.031 (0.806) 1.777 (<0.001) 1.879 (<0.001) 2.094 (<0.001)	1.171(0.249) 1.548(0.007) 1.540(0.020) 1.381(0.162)	1.290(0.020) 2.342(<0.001) 2.197(<0.001) 2.387 (<0.001)	0.747 (<0.001) 1.687 (<0.001) 2.680 (<0.001) 2.340 (<0.001)	0.544 (<0.001) 0.575 (<0.001) 0.865 (0.249) 1.921 (<0.001)	0.653 (0.001) 0.816 (0.180) 1.215 (0.241) 2.465 (<0.001)
Presence of chronic disease (baseline: No)	1.129 (0.220)	0.994 (0.956)	0.927 (0.352)	2.184 (0.005)	0.894 (0.180)	0.901 (0.265)
Inpatient treatment (baseline: zero treatment)	0.541 (<0.001)	0.505 (<0.001)	0.533 (<0.001)	0.516 (<0.001)	0.735 (0.002)	0.973 (0.769)
Urban (baseline: rural)	1.813 (<0.001)	1.600 (0.000)	1.679 (<0.001)	0.784 (<0.001)	1.369 (<0.001)	0.851 (0.087)
City (baseline: Xiamen) Beijing Shanghai	1.113 (0.282) 1.140 (0.195)	0.953 (0.642) 1.002 (0.984)	1.101 (0.268) 1.117 (0.206)	1.527 (0.548) 0.947 (0.823)	1.186 (0.047) 1.181 (0.056)	1.091 (0.343) 1.068 (0.478)

Numbers are “odds ratio (p-value)”. “Baseline” represents the reference group for OR calculation.

Broad coverage is needed for insurance to be effective. Analysis results presented above provide possible suggestions for future targets to raise coverage. Interesting target populations may include large households and households in a certain income range. It is of interest to note the negative association between coverage and inpatient treatment. Cost associated with inpatient treatment is an important component of catastrophic health expenditure [20], [21]. Insurance coverage needs to be raised as a prevention tool, or other policy interventions should be developed to protect households with inpatient treatments but no insurance. It is noted that the significant factors identified in our analysis were comparable to those in published studies.

### Analysis of gross medical cost

Medical cost had been rising significantly in China [22]. In the survey, household medical cost was designed as a categorical variable, which was easier to manage and less likely to be subject to recall error compared to a continuous variable. Two sets of analyses were conducted (Table 4).

**Table 4:** Medical cost: univariate and multivariate logistic regressions.

	Medical cost>1K				Medical cost>5K	
		Univariate	Multivariate	Univariate		Multivariate
Household size		1.139 (<0.001)	1.155 (<0.001)	1.124 (<0.001)		1.113 (0.003)
Income (baseline: <30K) B: between 30K and 50KC: between 50K and 100K D: between 100K and150K E: over 150K		1.191 (0.039) 1.376 (<0.001) 1.332 (0.002) 1.725 (<0.001)	1.229 (0.025) 1.359 (0.001) 1.210 (0.060) 1.808 (<0.001)	0.906 (0.467) 0.773 (0.063) 0.839 (0.262) 0.953 (0.771)		0.939 (0.664) 0.722 (0.025) 0.733 (0.057) 0.985 (0.931)
Presence of chronic disease (baseline: No)		2.735 (<0.001)	2.181 (<0.001)	2.524 (<0.001)		1.929 (<0.001)
Inpatient treatment (baseline: zero treatment)		3.777 (<0.001)	3.340 (<0.001)	3.658 (<0.001)		3.241 (<0.001)
Basic insurance		0.990 (0.928)	1.220 (0.092)	1.018 (0.920)		1.420 (0.069)
Commercial insurance		0.852 (0.119)	1.351 (0.016)	0.889 (0.499)		1.401 (0.107)
Urban (baseline: rural)		1.473 (<0.001)	1.413 (<0.001)	0.986 (0.895)		0.969 (0.790)
City (baseline: Xiamen) Beijing Shanghai		0.890 (0.089) 0.867 (0.038)	0.708 (<0.001) 0.806 (0.004)	0.959 (0.712) 0.939 (0.590)		0.814 (0.090) 0.920 (0.498)

Numbers are “odds ratio (p-value)”. “Baseline” represents the reference group for OR calculation.

In the first set, the contrasting groups were “medical cost >1 K Yuan” versus “≤1 K”. Among the 5,097 households, 3,019 (59.23%) were in the high-cost group. Multivariate analysis suggested that larger households tended to have higher medical expense, which was reasonable as the expense was not normalized by household size. Household income was significantly associated with medical expense. For example, with the lowest income group as reference, the highest income group had an odds ratio of 1.808 (p-value<0.001). This was partly caused by the lack of normalization. Both presences of chronic disease and inpatient treatment led to higher medical cost (odds ratios 2.181 and 3.340, respectively, both p-values<0.001). Basic insurance coverage was not significantly associated with medical expense. Commercial insurance coverage was significantly associated with medical expense (odds ratio 1.351, p-value 0.016). This significant positive association reflected its selection nature, with households in worse health conditions more likely to purchase commercial insurance. Urban residents had higher medical cost, as urban health care facilities tended to be more expensive. City was included as a covariate and found to be significant. In particular, both Beijing and Shanghai households tended to have lower expense, compared with Xiamen. The odds ratios for Beijing and Shanghai were similar (0.708 and 0.806, respectively, p-values <0.001 and 0.004).

In the second set of analysis, we contrasted low and moderate cost group (≤5 K Yuan) with the extremely high cost group (>5 K Yuan). Among the 5,097 households, 473 (9.28%) belonged to the extremely high cost group. Compared with the first set of analysis, the effect of household size was similar. The associations between income levels and expense were mostly insignificant, expect for the “between 50 K and 100 K” group (odds ratio 0.722, p-value 0.025). Presences of chronic disease and inpatient treatment were significantly associated with higher expense (odds ratios 1.929 and 3.241, respectively, p-values<0.001). Neither basic nor commercial insurance coverage rate was significant. The difference between urban and rural residents was not significant, and there was no significant difference among cities.

The fast rising of medical cost is a challenge encountered by not only China but also developed countries like the United States. Our analysis may assist identifying the factors that contributed to higher medical cost. As a limitation of this study and surveys of a similar kind, we were only able to identify the factors associated with cost, but not necessarily the underlying causal factors. Although there is no one-to-one correspondence between cost and quality of care, they tend to be correlated. An important aspect of basic health insurance is to ensure that service is delivered to all insured in an equal manner. From this perspective, our analysis identified populations with lower medical cost and possibly lower quality of care. From a policy point of view, for example, it is of interest to investigate how to make healthcare more affordable and more accessible to low-income group and people living in the rural area.

### Analysis of out-of-pocket medical cost

Gross medical cost can be of significant interest to the government and insurance industry. For households, net out-of-pocket cost – medical cost after insurance reimbursement – is of more importance. It provides a more direct measure of the impact of illness conditions and effect of medical insurance. It has been observed that in several Asian countries, high out-of-pocket cost has been an important contributing factor for poverty [18]. As such, an important goal of China's health reform was to reduce out-of-pocket cost [19]. We removed households with zero coverage, as we were interested in the impact of health insurance. 5,070 out of 5,097 households were included in analysis (Table 5).

**Table 5:** Out-of-pocket medical cost: univariate and multivariate logistic regressions.

	Medical cost>1K		Medical cost>5K	
	Univariate	Multivariate	Univariate	Multivariate
Household size	1.170 (<0.001)	1.198 (<0.001)	1.043 (0.227)	1.100 (0.020)
Income (baseline: <30K) B: between 30K and 50K C: between 50K and 100K D: between 100K and150K E: over 150K	1.206 (0.032) 1.390 (<0.001) 1.236 (0.027) 1.718 (<0.001)	1.296 (0.006) 1.376 (<0.001) 1.105 (0.334) 1.759 (<0.001)	1.172 (0.292) 0.781 (0.126) 0.806 (0.239) 1.140 (0.475)	1.377 (0.041) 0.811 (0.210) 0.757 (0.142) 1.209 (0.320)
Presence of chronic disease (baseline: No)	2.635 (<0.001)	2.175 (<0.001)	2.006 (<0.001)	1.751 (<0.001)
Inpatient treatment (baseline: zero treatment)	3.287 (<0.001)	2.743 (<0.001)	2.702 (<0.001)	2.489 (<0.001)
Basic insurance	0.860 (0.175)	1.054 (0.660)	1.036 (0.868)	1.391 (0.134)
Commercial insurance	0.875 (0.204)	1.476 (0.003)	1.931 (<0.001)	2.977 (<0.001)
Urban (baseline: rural)	1.317 (<0.001)	1.237 (0.005)	0.805 (0.061)	0.681 (0.003)
City (baseline: Xiamen) Beijing Shanghai	1.276 (<0.001) 1.240 (0.003)	1.115 (0.150) 1.234 (0.006)	1.380 (0.015) 1.354 (0.023)	1.254 (0.098) 1.353 (0.028)

Numbers are “odds ratio (p-value)”. “Baseline” represents the reference group for OR calculation. Sample size  = 5070.

In the analysis of low (≤1 K Yuan) versus high (>1 K Yuan) cost, household size was significant, with larger households more likely to have higher cost (odds ratio 1.198). With income <30 K as reference, three higher income levels were significantly associated with higher cost. The only insignificant level was “between 100 K and 150 K”. Presences of chronic disease and inpatient treatment were significantly associated with higher cost (odds ratios 2.175 and 2.743, respectively, p-values<0.001). This result suggested that health insurance was not able to fully remove the financial burden caused by illness. Similar observations had been made in recent studies conducted in South Korea and Vietnam [16], [18]. The association for basic insurance coverage was not significant (p-value 0.660), whereas it was significant for commercial insurance (odds ratio 1.476, p-value = 0.003). Wagstaff and Lindelow [23] suggested that such an observation could be explained by supplier-induced demand. In the literature, there were conflicting observations on the association between out-of-pocket cost and insurance coverage in China. For example, Wagstaff and Lindelow [23] reported a positive association, whereas Xiao and others [24] and Wagstaff and others [25] reported negative associations. Note that those three studies all focused on NCMS, which covered rural areas. Our analysis might provide insights into the association for urban and surrounding residents. There was a significant difference between urban and rural areas, with urban residents paying more out-of-pocket cost (odds ratio 1.237). This could be explained by the higher quality of care in urban areas. Residents in Shanghai paid significantly more out-of-pocket cost than Xiamen residents (odds ratio 1.234). For covariates overlapped with those in [19], the qualitative findings were mostly consistent.

Results from the comparison of low and moderate cost (≤5 K Yuan) versus extremely high cost (>5 K Yuan) were comparable to those described above. Household size remained significant. However, most associations between income levels and cost were not significant. Only the “between 30 K and 50 K” group showed a significant higher level of cost, compared with the reference group. Presences of chronic disease and inpatient treatment remained significant, with the odds ratios slightly lower than those for the gross cost. Basic insurance was not significant, whereas a higher commercial insurance coverage rate was positively correlated with higher cost (odds ratio 2.977). Urban residents tended to have a lower probability of extremely high out-of-pocket cost. A possible explanation was that the reimbursement system in urban areas was more developed, leading to a higher amount and percentage of reimbursement and hence a lower probability of extremely high cost. In addition, the urban basic health insurance system had a better coverage for catastrophic expense. Note that this result needs to be interpreted cautiously, as only 137 records fell in the category of “rural residents and extremely high out-of-pocket cost”. Residents of Shanghai had a higher probability of having extremely high cost (odds ratio 1.353, p-value 0.028), compared to Xiamen.

### Analysis of coping strategies

With a considerable amount of out-of-pocket cost, the means that households pay for the cost are of interest. In the survey, the interviewees were asked what was the most important financial source to pay for out-of-pocket cost, with answers including (A) salary from last month, (B) saving, (C) help from family and friends, (D) loan, and (E) reducing daily living cost. In the whole cohort, percentages of answering (A)–(E) were 60.53%, 34.06%, 0.57%, 0.65% and 4.20%, respectively. The majority of the households were able to self-finance out-of-pocket cost (answers A and B). Two sets of analyses were conducted (Table 6). In the first set, we compared strategies (A) versus (B)–(E). Covering medical cost using last month's salary was the “best” coping strategy, imposing the least long term impact. In the second set of analysis, we compared strategies (A)–(B) versus (C)–(E), as (A) and (B) corresponded to self-finance.

**Table 6:** Analysis of coping strategy: univariate and multivariate logistic regressions.

	Other than “Salary”	Other than “Salary + Saving”		
	Univariate	Multivariate	Univariate	Multivariate
Household size	1.001 (0.944)	0.969 (0.171)	0.978 (0.601)	0.906 (0.051)
Income (baseline: <30K) B: between 30K and 50K C: between 50K and 100K D: between 100K and150K E: over 150K	0.661 (<0.001) 0.839 (0.030) 0.819 (0.031) 0.894 (0.265)	0.674 (<0.001) 0.816 (0.019) 0.732 (0.001) 0.835 (0.094)	0.447 (<0.001) 0.306 (<0.001) 0.341 (<0.001) 0.082 (<0.001)	0.656 (0.003) 1.637 (<0.001) 0.490 (<0.001) 0.335 (<0.001)
Presence of chronic disease (baseline: No)	0.864 (0.028)	0.770 (<0.001)	1.813 (<0.001)	0.101 (<0.001)
Inpatient treatment (baseline: zero treatment)	1.314 (<0.001)	1.353 (<0.001)	1.606 (<0.001)	1.499 (0.004)
Basic insurance	0.976 (0.824)	1.002 (0.988)	0.715 (0.129)	0.972 (0.901)
Commercial insurance	0.968 (0.748)	0.722 (0.010)	0.629 (0.050)	0.528 (0.027)
Urban (baseline: rural)	0.805 (<0.001)	0.763 (<0.001)	0.599 (<0.001)	0.357 (<0.001)
City (baseline: Xiamen) Beijing Shanghai	3.016 (<0.001) 3.047 (<0.001)	3.130 (<0.001) 3.193 (<0.001)	0.995 (0.969) 0.965 (0.809)	1.095 (0.555) 1.089 (0.585)

Numbers are “odds ratio (p-value)”. “Baseline” represents the reference group for OR calculation. Sample size  = 5070.

In the first set of analysis, we coded the outcome variable “using salary” as 0 and “using other means” as 1. Table 6 suggested that household size was not an important factor in deciding coping strategies. The influence of income was significant, with higher income groups less likely to pursue means other than salary (odds ratios 0.674, 0.816, 0.732, and 0.835, respectively). However, there was a lack of linear relationship (p-value<0.001). This result showed that high-income households were able to cover out-of-pocket medical cost with regular income, without having to suffer any long term financial impact from illness. The odds ratio for the presence of chronic disease was 0.770 (p-value<0.001). Chronic diseases are recurrent, with low to moderate cost for each episode. Well-planned households usually have well-adjusted coping plans that can cover cost using monthly income without having to resort to outside financial sources. The odds ratio for the presence of inpatient treatment was significant (1.353, p-value<0.001). Inpatient treatments happen with low frequencies and hit households “without warning”. As it was difficult to plan for such incidents ahead, households were more likely to pursue coping strategies other than salary. The effect of basic insurance was not significant, while the effect of commercial insurance was (odds ratio 0.722, p-value 0.010). This suggested the commercial insurance's positive effect on eliminating the long-term financial impact of illness. Urban residents were more likely to use salary (p-value<0.001), which was explained by the higher salary income of urban residents as well as the cultural difference between urban and rural China. Both residents of Beijing and Shanghai had a higher likelihood of pursuing coping strategies other than salary.

In the second set of analysis, we coded the outcome variable “salary or saving” as 0 and “other means” as 1. Table 6 showed that although there were some quantitative differences, the qualitative conclusions were similar to the first set of analysis. Notable differences included income level “between 50 K and 100 K”, which had an odds ratio greater than 1 (1.637, p-value<0.001), showing that this group was more likely to pursue other coping strategies than the reference group. Another difference was that for both Beijing and Shanghai, the difference from Xiamen was not significant.

The majority of surveyed households were able to self-finance out-of-pocket medical cost using salary and saving, without having to rely on outside financial sources or reducing daily living cost. However, there were still 5% of the households that warranted further attention. Future policy development should focus on this group that may suffer a long financial impact caused by out-of-pocket medical cost.

### Limitations

Investigating both basic and commercial insurance might provide a more comprehensive description of households’ insurance status. However, a tradeoff is that the policy implications of the study was less lucid as the *net* effect of basic insurance could not be investigated – it is thus not clear how the government should tune the basic insurance policy.

Another limitation was that because of limitation in data collection, we were not able to fully validate the representativeness of samples. Summary statistics on the household size and income data roughly matched data published by the central government on the three cities. However, as some data (e.g. income) were collected using categorical variables, we were not able to conduct a rigorous test on the representativeness.

In the survey, all the samples were drawn from three major cities and surrounding areas. As can be seen from the GDP figures and city locations (Table 1), the samples were not representative of the Chinese population. However, as it is estimated that at least 60% of Chinese population live within 400 kilometers of the east coastline, our study may still be of significant value. Nevertheless, a counterpart study focusing on the poorer, more remote rural areas should be pursued in future studies.

With phone call surveys, we were able to collect a large number of samples at affordable cost. However, the data collected were inevitably less detailed. Particularly, the data were either snapshots at the time of survey or aggregated data over twelve months. Such data had limitations. For example, the insurance status (particularly for commercial insurance) and household size might change over time. The aggregated data, including inpatient treatment, presence of chronic disease, out-of-pocket cost and coping strategies, could not describe variations across different disease episodes and their differences in financial consequences. In addition, measuring out-of-pocket cost as a single item might result in a biased estimation (usually under-estimation; [26]). On the other hand, the present design might also have advantages. Particularly, households often had multiple illness episodes, and people tended to have a better recollection of the total cost and how they paid for all of them in general, rather than for a single episode. It was possible or even likely that multiple coping strategies were taken, while we focused on the *most important* coping strategy.

In computing summary statistics and regression analysis of coverage, we dichotomized the coverage rate at 50% and created binary variables. As discussed above, there was no well-developed approach for analyzing percentage data with non-ignorable corners. We experimented with a few other cutoffs (e.g. 80%) and reached similar conclusions.

### Conclusions

The healthcare sector in China is undergoing tremendous reform. Its development may provide valuable information for reform in other developing countries. In this study, we reported a phone call survey in three major cities. Our findings suggested that the surveyed population were well covered by basic and commercial health insurance, although there was still room for improvement. Possible target subpopulations to further increase coverage were identified. This study also identified factors that contributed to high gross and net out-of-pocket medical cost. More attention should be paid to these factors in the process of reform. We also identified the group that had to cope with out-of-pocket medical cost by borrowing or reducing daily living cost. Policy interventions should be developed targeting that group. Despite several limitations, this study may provide valuable information on the current status of China's health insurance and serve as basis for future policy development.
